# Supplementary material for: Developing programme theory for a place-based, systems change approach to adolescent mental health: A developmental realist evaluation
Source: PLOS Ment Health. 2025 Jun 9;2(6):e0000226. doi: 10.1371/journal.pmen.0000226 (PMC12798369; doi:10.1371/journal.pmen.0000226)
Supplement: S1 Text — (DOCX) [file pmen.0000226.s001.docx]

**Description of and rationale for realist approach**

Realist evaluation is a theory-driven approach that explores how causal mechanisms and contexts interact and lead to outcomes [intended or unintended] [1]. The realist approach is grounded in the realist philosophy of science, which asserts that mechanisms can be identified within the social systems in which they operate [2, 3]. These mechanisms describe how a programme’s resources or opportunities influence the reasoning of individuals and produce behaviour change. This process can be either enabled or constrained by wider factors or ‘contexts’ [4].

The realist approach works from the assumption that programmes are ‘theories incarnate’; when a programme is developed, even though it may not be explicit, there are one or more underpinning theories about what might lead to change [3]. Realist evaluation seeks to make a programme’s underlying theories explicit through the development of clear hypotheses [or ‘initial programme theories’] about how, for whom and in which contexts the intervention might create change. These theories are then tested and iteratively refined in response to emerging changes in the system, making the realist evaluation approach particularly well-suited to evaluating complex interventions [5].

This realist developmental evaluation approach allowed our research team to address two critical objectives in the Kailo framework development and evaluation (see [6, 7]): the need for formative evaluation in the early stages of the Kailo programme to build the theory behind the framework prior to an impact/outcome evaluation, and the need for a greater focus and understanding of context during initial delivery and effective scale-up Kailo.

*Table 1. Definition of context, mechanism and outcome (adapted from Jagosh, Bush [8])*

| **Realist term** | **Definition** |
| --- | --- |
| Context | The “backdrop” of programmes and research. Contexts (C) could include things like cultural norms, history of a community, local capacity, geographic location (e.g., rural or urban), existing social networks, local policy, funding or infrastructure. Programme resources or activities (see below) may change the context during programme implementation, with contexts operating in time. |
| Mechanism | Made up of two, interacting parts: the programme resource (referred to in this paper as ‘MResO’) and the ways in which participants interpret or respond (or do not respond) to this resource (referred to in this paper as ‘MResP’) [9]. Mechanisms are a generative force that lead to outcomes in a specific context. In this developmental evaluation, mechanisms may refer to why community stakeholders recognise the Kailo programme as having value (or not) or why they are enthusiastic to engage in the programme (or not). |
| Outcome | Broader than the goals of a given programme, outcomes (O) can be intended or intended and may refer to immediate, intermediate, or final outcomes. Outcomes from one CMOC may, over time, become the context for new activities or mechanisms. This is described as a ‘ripple effect’ [8]. |

**References**

1. Pawson R. The Science of Evaluation: A Realist Manifesto. London: Sage.; 2013.

2. Bhaskar R. A Realist Theory of Science: Routledge; 2013 2013/1//.

3. Wong G, Westhorp G, Manzano A, Greenhalgh J, Jagosh J, Greenhalgh T. RAMESES II reporting standards for realist evaluations. BMC Medicine. 2016;14(1):96-.

4. Pawson R, Tilley N. Realistic Evaluation. London: Sage; 1997.

5. Shearn K, Allmark P, Piercy H, Hirst J. Building Realist Program Theory for Large Complex and Messy Interventions. International Journal of Qualitative Methods. 2017;16(1).

6. Brand SL, Quinn C, Pearson M, Lennox C, Owens C, Kirkpatrick T, et al. Building programme theory to develop more adaptable and scalable complex interventions: Realist formative process evaluation prior to full trial. Evaluation. 2019;25(2):149-70.

7. Bamberger M, Vaessen J, Raimondo E. Chapter 1: Complexity in development evaluation In: Bamberger M, Vaessen J, Raimondo E, editors. Dealing with complexity in development evaluation: a practical approach. London: Sage; 2016.

8. Jagosh J, Bush PL, Salsberg J, Macaulay AC, Greenhalgh T, Wong G, et al. A realist evaluation of community-based participatory research: partnership synergy, trust building and related ripple effects. BMC Public Health. 2015;15(1):725.

9. Dalkin SM, Greenhalgh J, Jones D, Cunningham B, Lhussier M. What’s in a mechanism? Development of a key concept in realist evaluation. Implementation Science. 2015;10(1):49.
