# Supplementary material for: Developing programme theory for a place-based, systems change approach to adolescent mental health: A developmental realist evaluation
Source: PLOS Ment Health. 2025 Jun 9;2(6):e0000226. doi: 10.1371/journal.pmen.0000226 (PMC12798369; doi:10.1371/journal.pmen.0000226)
Supplement: S2 Text — (DOCX) [file pmen.0000226.s002.docx]

**Key Kailo terms and stakeholders**

| **Key Kailo terms** | **Definition** |
| --- | --- |
| Early Discovery | Involved activities to develop understanding of the local context, including fostering relationships and partnerships with local community stakeholders, identifying needs and opportunities, and forming communities around shared priorities. |
| Deeper Discovery and Co-design | Involved **co-design sessions** (otherwise known as ‘**small circle sessions**’) with young people and community professionals to design solutions and strategies to address the priorities identified in Phase 1. This phase also included ‘**big circle sessions**’ where the Kailo delivery team fed back learning from the co-design sessions to a wider group of community stakeholders (including community professionals and system leaders) who were also asked to contribute to the work. |
| Implementation and testing | Focused on the process of embedding and sustaining the strategies developed in Phase 2. |
| **Key Kailo people** | **Definition** |
| Kailo consortium | The Kailo consortium is made up of academics, designers, and practitioners who are working on Kailo in Newham and Northern Devon. |
| Kailo delivery team | The Kailo delivery team is part of the Kailo consortium (see above) and is responsible for delivering the work (i.e., working with communities, conducting co-design sessions etc.) in Newham and Northern Devon. |
| Kailo facilitator | Kailo facilitators are people part of the delivery team who are responsible for facilitating co-design sessions (i.e., small circle sessions). |
| Kailo evaluation team | The Kailo evaluation team is part of the Kailo consortium (see above) and is responsible for the evaluation of Kailo in Newham and Northern Devon. |
| Kailo community researchers | Young people aged 16-25 living in the local area who are employed by Kailo to support with the delivery of the programme in Newham and Northern Devon. |
| Kailo Young Person’s Advisory Groups (YPAGs) | The Kailo YPAGs were set up to support the Kailo evaluation team in the developmental evaluation of Kailo. |
| Community stakeholders | Includes any member of the community from young people, parents, other adults in a non-professional capacity, adults in a professional capacity. |
| Young people | Young people who have been involved in the co-design sessions (i.e., small circle sessions). |
| Community professionals | Community professionals include those who have been involved in either the co-design sessions (i.e., small circle sessions) and/or wider big circle sessions. |
| Senior leadership | Senior leadership includes those who have decision making power within the community. |
| Adults | Any adults (e.g., community professionals, Kailo delivery team) present in the co-design sessions. |
