# Supplementary material for: Developing programme theory for a place-based, systems change approach to adolescent mental health: A developmental realist evaluation
Source: PLOS Ment Health. 2025 Jun 9;2(6):e0000226. doi: 10.1371/journal.pmen.0000226 (PMC12798369; doi:10.1371/journal.pmen.0000226)
Supplement: S3 Text — (DOCX) [file pmen.0000226.s003.docx]

**Types of activities observed as part of the developmental evaluation**

| **Kailo activities, processes, and meetings** | **Frequency** | | **Description** |
| --- | --- | --- | --- |
|  | **Newham** | **Northern Devon** |  |
| Newham and Northern Devon delivery team meetings | Semi-regular  (one in August 23 and four between Jan – May 24) | Weekly from Jul 23 – Jun 24 | Site-specific team meetings to discuss progress and plans for next sessions. |
| Delivery team co-ordination meetings | Bi-weekly from Jul 23 – Jun 24 | | WS1 team meetings to reflect on progress and discuss and resolve any challenges in both sites. |
| Small circle co-design sessions | 14 sessions for A4W and 15 for V&C group between  Sep 23 – Jun 24 | 15 sessions for each group between Jul 23 – Mar 24 | Co-design sessions conducted with young people and relevant community professionals to design strategies to address identified opportunity areas. |
| Big circle sessions | One session between in January 2024 | Three sessions between Sep 23 – Mar 24 | Sessions with a wider group of local system leaders and community professionals to feedback on small circle session progress and further shape design ideas. |
| Community partner meetings | Three sessions between Sep 23 – Jun 24 | 11 sessions between May 23 – Jun 24 | Sessions with local community professionals involved in the delivery or work of the small circle sessions to update on progress and problem solve any challenges faced. |
| Delivery team away day | One session in Apr 24 | | Whole delivery team away days designed to reflect on progress, problem solve challenges, and share learning. |
