# Supplementary material for: Developing programme theory for a place-based, systems change approach to adolescent mental health: A developmental realist evaluation
Source: PLOS Ment Health. 2025 Jun 9;2(6):e0000226. doi: 10.1371/journal.pmen.0000226 (PMC12798369; doi:10.1371/journal.pmen.0000226)
Supplement: S4 Text — (DOCX) [file pmen.0000226.s004.docx]

**Round 1 interview schedule example – Kailo consortium**

**INTRODUCTORY QUESTIONS**

1. Can you tell me about your role in Kailo?

Probes:

How long have you been involved?

What are your main responsibilities?

How has your role evolved?

**PROGRAMME OUTCOMES - EARLY DISCOVERY**

1. What do you think were the most important outcomes of the *early discovery phase* for **young people**?
2. To what extent were the outcomes of the *early discovery phase* the same for all **young people**?

Probes:

Why/how might they be different?

What about [insert subgroups]?

1. What do you think were the most important outcomes of the *early discovery phase* for **local stakeholders/system leaders**?
2. To what extent were the outcomes of the *early discovery phase* the same for all **local stakeholders/system leaders**?

Probes:

Why/how might they be different?

What about [insert subgroups]?

**PROGRAMME OUTCOMES - DEEPER DISCOVERY/CO-DESIGN**

1. What do you think will be the most important outcomes of the *deeper discovery/ co-design phase* for **young people**?
2. To what extent do you think the outcomes of the *deeper discovery/co-design phase* will be the same for all **young people**?

Probes:

Why/how might they be different?

What about [insert subgroups]?

1. What do you think will be the most important outcomes of the *deeper discovery/ co-design phase* for **local stakeholders/system leaders**?
2. To what extent do you think the outcomes of the *deeper discovery/co-design phase* will be the same for all **local stakeholders/system leaders**?

Probes:

Why/how might they be different?

What about [insert subgroups]?

**PROGRAMME MECHANISMS**

1. We are curious about how Kailo achieves its outcomes. How do you think the programme will achieve or help to achieve [*outcome*]?

Probes:

What are the contributions of Kailo’s activities and resources?

How might the co-design sessions help Kailo achieve its outcomes?

Research expertise

Service design expertise

1. Is Kailo providing anything that is new? What/how/why?
2. How do you think Kailo might change the way that **system leaders and local stakeholders** think or feel about addressing the social determinants of adolescent mental health? Can you provide examples?
3. There are lots of ideas about how Kailo works, and we think it might work differently in different places for different people. One of these ideas is through building partnerships and trust in the community. Has it worked like this for you? Can you give an example?

Another idea is that Kailo works by integrating co-design approaches with academic evidence. Has it worked like this for you? Can you give an example?

**PROGRAMME CONTEXTS**

1. We’ve seen that Kailo works differently in different places. What is it about Newham/North Devon that makes it work more or less effectively?

Probes:

How has the **local culture** influenced the success of Kailo?

How have **local resources** influenced the success of Kailo?

How have **local relationships** influenced the success of Kailo?

1. What factors have **supported/inhibited** the implementation of Kailo?

Probes:

What are the main **challenges/facilitators** in **Newham/North Devon**?

What are the main **challenges/facilitators** in the **Kailo consortium**?

**CONCLUDING QUESTIONS**

1. If you could change something about Kailo to make it work more effectively, what would you change? Why?
2. What else do you think we need to know to understand how Kailo works in Newham/North Devon?
3. Who else should we speak to (in Newham/North Devon/the consortium) about Kailo? Can you put us in contact?
