# Supplementary material for: Developing programme theory for a place-based, systems change approach to adolescent mental health: A developmental realist evaluation
Source: PLOS Ment Health. 2025 Jun 9;2(6):e0000226. doi: 10.1371/journal.pmen.0000226 (PMC12798369; doi:10.1371/journal.pmen.0000226)
Supplement: S5 Text — (DOCX) [file pmen.0000226.s005.docx]

**Round 2 interview schedule example – Kailo consortium**

**Introductory questions**

1. Can you tell me about your role on Kailo?
   1. How long have you been involved?
   2. What are your main responsibilities?
   3. How has your role evolved?

**Understanding of local community**

1. (**1.2., 4.3**) Previous research has suggested that developing an understanding of the local community is important for programmes like Kailo. Has this been your experience?
   1. (**1.2, 4.3**) Can you give me an example of the things you have done in [site] to develop an understanding of the local context?
   2. (**1.2, 4.3**) Has this been helpful in any way? Can you tell me why this has been helpful? What is it about understanding the local community that is important? **Prompts:** (C) cultural differences, previous experiences in [site], (M) deeper understanding of everyone’s view, allows to build on strengths, improves confidence of community members.
   3. (**1.2, 4.3**) What impact do you think having this understanding of [site] has had? **Prompts:** establish trust and meaningful connection, begins to address power imbalances, greater engagement by community stakeholders.
2. (**1.5**) The literature suggests that taking a flexible approach to planning and programme development is important in programmes like Kailo. Is this something you relate to?
   1. (**1.5**) What impact does taking a flexible approach have? We were thinking it helps create the opportunity for community voices to be heard, which can help make the programme more responsive to community needs and enables the programme to embed change within the community.
   2. (**1.5**) What is needed to enable you to take this flexible approach to planning and programme development? e.g., money, time...

**Creating a safe co-design space**

1. (**1.1**) We know that it is really important for co-design spaces to feel non-judgemental and accessible. Can you tell me about how you have created this non-judgemental and accessible co-design space in [site]? **Prompts:** thinking carefully about language and inclusion, promoting equity and inclusion.
   1. Are there particular qualities a person needs to be enable them to do this? **OR** What enabled you to do this? **Prompts:** aware of the importance of creating safe and validating spaces.
   2. Can you tell me about why it might be important to create a non-judgemental and accessible space for small circle sessions? We thought it might help young people be open and honest about their experiences.

**Social determinants**

1. (**ALL**) One of the key things about Kailo is taking a social determinants lens to preventing adolescent mental ill-health. Can you tell me about your understanding of social determinants?
   1. How have you created this focus on social determinants in [site]?
   2. What has enabled you to do this?
   3. What impact has having this ‘social determinants’ lens had on the work in [site]?

1. (**ALL**) Where there is this constant pull towards individualistic ways of thinking (i.e., individual problems and solutions), it can be quite hard to maintain this focus on social determinants. Do you relate to this?
   1. What makes it a challenge to maintain this focus on social determinants?
   2. What would help better maintain this focus on social determinants? We were thinking things like the naming of the opportunity area, narrowing the focus on specific solutions, using evidence, and community partner clarity on roles might be important.

1. (**ALL**) We were thinking that the framing of the opportunity area might be important for maintaining the focus on social determinants.  What do you think about this?
   1. Has the naming of the opportunity area as ‘activities for wellbeing’ helped or hindered this focus on social determinants? Why?

1. (**ALL**) We are interested to know what impact this focus on social determinants has had for young people and community partners.
   1. What do you think young people got out of Kailo’s focus on social determinants? **Prompts:** empowered, think differently about MH.
   2. What do you think community partners got out of Kailo’s focus on social determinants?

**Ownership**

1. (**BROAD**) We are interested in how communities might take ownership over the strategies developed and the Kailo framework.
   1. How willing do you feel the community is to take ownership over the strategies developed in [site]? What has helped/hindered this willingness to take ownership?
   2. How willing do you feel the community is to take ownership over the Kailo framework? What has helped/hindered this willingness to take ownership?

**End**

1. We know that Kailo will look a little different moving forward. For example, there won’t be a Kailo consortium and the framework will be community led. With this in mind, if you could change something about Kailo to make it work more effectively, what would you change? Why?

1. What else do you think we need to know to understand how Kailo works in [site]?
