# Supplementary material for: Developing programme theory for a place-based, systems change approach to adolescent mental health: A developmental realist evaluation
Source: PLOS Ment Health. 2025 Jun 9;2(6):e0000226. doi: 10.1371/journal.pmen.0000226 (PMC12798369; doi:10.1371/journal.pmen.0000226)
Supplement: S6 Text — (DOCX) [file pmen.0000226.s006.docx]

**Initial CMOCs**

**1. Building relationships and trust**

**1.1** When adults are aware of the importance of creating validating and safe spaces (C), facilitators thinking carefully about language and inclusion (MResO) can make the space feel non-judgemental and accessible (MResP), helping young people to be open and honest about their experiences (O).

**1.2** Where adults are aware of the importance of creating validating and safe spaces (C), adapting co-design sessions to promote equity and inclusion (MResO) can make the space feel non-judgemental and accessible (MResP). This increases young people’s engagement in the sessions (O).

**1.3** Where there are cultural differences between the programme team and local communities (C), the programme team engaging in a community’s way of knowing and demonstrating reflexivity (MResO) can lead to a deeper understanding of everyone’s worldview (MResP). This can help to establish trust and meaningful connection between the team and communities (O1) and may begin to address power imbalances (O2).

**1.4** Where new programmes are introduced (C), the programme team visibly involving trusted/recognised members of the community in programme activities (MResO) means stakeholders recognise the programme as having value (MResP). This can lead to greater engagement in the work (O).

**1.5** Where external programmes are long-term (C), having sufficient time for the initial stages (MResO) creates space for the programme team to understand the local ecosystem (MResP). This can facilitate the building of trusting relationships between the programme team and local community members (O).

**1.6** Where programmes have sufficient time (C1) and programme teams are open to learning and adapting (C2), taking a flexible approach to planning and programme development (MResO) provides the opportunity for community voices to be heard (MResP). This allows for the programme to be more responsive to community needs (O1) and to embed change within the community (O2).

**1.7** Where multiple different organisations come together for the first time (C), programme teams facilitating the development of a shared vision (MResO) can help create a collective understanding of the issue (MResP) and may lead to a stronger foundation for partnership working (O).

**1.8** Where multiple organisations work in siloes and have limited opportunities to work together around a particular challenge (C) programme teams bringing organisations together for the first time and facilitating the development of a shared vision (MResO) can help create a collective understanding of the issue (MResP). This leads to a stronger foundation for partnership working (O).

**1.9** Where multiple different organisations come together for the first time (C), the programme team facilitating the prioritisation of locally situated issues (MResO) can help create a collective energy to address the prioritised issue (MResP). This leads to greater commitment from community members to work with the team in the following phases (O).

**2. Bringing a social determinants lens**

**2.1** Where organisations and services are often focused on an individual model of mental health (C), programmes bringing an explicit focus on social determinants (MResO) can shift understanding to include the wider environment (MResP) and help community partners/professionals think more holistically about young people’s mental health (O).

**2.2** Where there is a central team who have a shared understand of social determinants (C), employing a social determinants lens/prioritising social determinants (MResO) can help orientate local stakeholders towards a different way of thinking about mental health (MResP). This helps to ensure local stakeholder efforts are focused on addressing the drivers of adolescent mental health (O).

**2.3** Where there are varying perspectives around what drives/influences young people’s mental health (C), building a locally situated social determinants framework in collaboration with the local community (MResO) can help the team feel clear and confident on the boundaries of the work (MResP). This helps to create and maintain a focus on the key drivers surfaced and prioritised by young people (O).

**2.4** Where there is a constant pull towards individualistic ways of thinking (C), framing the opportunity area in a way that aligns with a social determinant (MResO) can help the co-design participants maintain shared understanding of the focus and boundaries of the work (MResP). This enables local stakeholders to focus on specific areas of change that can improve adolescent mental health in the longer term (O).

**2.5** In the context of a safe, trusted space (C), training young people in structural determinants of mental health (MResO) opens their eyes to wider systemic issues (MResP) and helps to create a collective understanding of their experience (O).

**2.6** Where public understanding of mental health is dominated by biomedical/individualistic concepts (C), training young people on the social determinants of mental health through activities like ‘system mapping’ (MResO) allows them to develop a better understanding of their own lived experience (MResP) and empowers them to take shared control over design ideas (O).

**2.7** Where the programme team bring a new understanding of structural determinants (C), conversations about social determinants and/or root causes (MResO) can lead to increased confidence and communication of needs, and better understanding of lived experience beyond symptoms (MResP). This helps young people (or community stakeholders) feel empowered to take action (O).

**2.8** In the context of limited knowledge (C), mapping local community power relations (MResO) enables informed decision making about who to involve (MResP) so that local leaders/decision makers are engaged in the programme (O).

**3. Empowering young people and community stakeholders**

**3.1** Where young people are the focus of a new programme (C), involving young people from the early stages of design/planning (MResO) means that young people are able to shape designs (MResP) and make the output more relevant (O).

**3.2** Where adults are committed to centring youth voice (C), training in participation models and facilitation (MResO) improves confidence and clarity of roles (MResP), enabling them to employ strategies to create safe and empowering spaces for young people (O).

**3.3** When meaningful youth voice is present in co-design sessions (C), modelling the co-design process with young people (MResO) helps community partners develop their skills in facilitation (MResP) and they may feel empowered to use these activities in their daily work (O).

**3.4** Where community partners have limited experience of involving young people in decision making (C), the programme team creating spaces where community partners and young people can come together (MResO) can help community partners better understand the value of young people’s perspectives (MResP). This leads community partners to think more about how they can involve young people in their work (O).

**3.5** Where system leaders have limited experience of engaging youth in strategy design but are aware of the increasing focus on youth voice (C), giving them opportunities to see the outputs from the co-design sessions with young people (MResO) means they may see the value of youth voice (MResP) and endorse the work Kailo is doing (O1) and shift their practice to include young people in mental health strategy design (O2).

**3.6** When young people are already interested and engaged in the topic of the opportunity areas (C), adults creating safe and empowering spaces (MResO) helps young people to feel useful (MResP) and increases their motivation to take part in sessions (O).

**3.7** When young people are uninspired in school/work/life (C), giving them the opportunity to learn from adults and peers in co-design sessions (MResO) inspires young people (MResP), meaning that young people are motivated to attend sessions (O1) and engage in the co-design work (O2).

**3.8** ...paying young people to attend co-design sessions (MResO) can make young people feel valued (MResP). This leads to increased motivation to attend co-design sessions (O).

**4. Ownership and sustainability**

**4.1** An understanding of a community’s past experience of engagement/involvement in initiatives (C) allows programme teams (Kailo) to build on strengths and avoid duplication (MResO) improving confidence among community members in the possibility of systemic change (MResP) and lead to greater engagement by community stakeholders (O).

**4.2** Communities with limited past research involvement (C), are more enthusiastic about the opportunity to take part in change efforts (M), leading to greater engagement from relevant community members (O).

**4.3** When people have no experience of effecting change in their community (C), demonstrating concrete changes to local strategies (MResO) creates belief among co-design participants that system change is possible (MResP). This leads to greater motivation and commitment to the work (O1) and conversations around how they can create change themselves, outside of Kailo (O2).

**4.4** ... (C), if the Kailo programme team actively involve system leaders at all stages (MResO), then they will be more willing to take ownership of the Kailo framework (MResP), and this could lead to greater likelihood of continuing Kailo framework activities (e.g. re-discovery) (O).

**4.5** ... (C) the Kailo team actively involving community partners at all stages of the co-design (MResO) leads to community partners taking ownership over the co-designed strategies (MResP). This means community partners are likely to advocate for the strategies to be adopted in the local community (O).

**4.6** When facilitators are committed to co-design (C), involving young people in all stages of the design process (MResO) can lead to young people feeling proud of the co-designs (MResP). This leads to young people taking a sense of ownership over what is developed (O).
