# Supplementary figures and images for: Developing programme theory for a place-based, systems change approach to adolescent mental health: A developmental realist evaluation
Source: PLOS Ment Health. 2025 Jun 9;2(6):e0000226. doi: 10.1371/journal.pmen.0000226 (PMC12798369; doi:10.1371/journal.pmen.0000226)

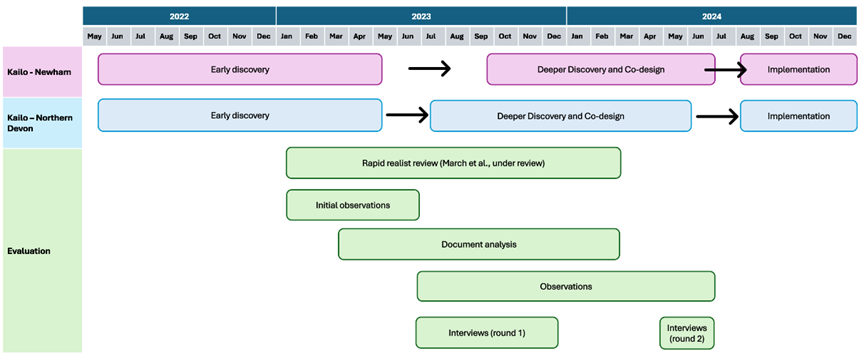

Supplement: S1 Fig — (TIF) [file pmen.0000226.s007.tif]
