# Supplementary material for: Developing programme theory for a place-based, systems change approach to adolescent mental health: A developmental realist evaluation
Source: PLOS Ment Health. 2025 Jun 9;2(6):e0000226. doi: 10.1371/journal.pmen.0000226 (PMC12798369; doi:10.1371/journal.pmen.0000226)
Supplement: Attachment — Submitted filename: Response to Reviewers - PLoS Mental Health.docx [file pmen.0000226.s009.docx]

University of Exeter

2.05c South Cloisters

St Luke’s Campus

Heavitree Road

Exeter

EX1 2LU

17^th^ April 2025

Dear Editor and Reviewers,

**Reviewer #1:**

Thank you so much for taking the time to read and review our manuscript. We are really pleased that you agree the topic is important and we are grateful for all of your comments, which we respond to in turn below.

| **Comment** | **Response** |
| --- | --- |
| It would be brilliant if the authors could add a diagram which demonstrated the process undertaken in the development evaluation, and how each step fit in within broader Kailo program stages and objectives. It was sometimes hard to follow what was happening and a diagram could help guide participants' understanding. | Thank you, there is a figure in the appendices (C) which shows the different phases of Kailo and the evaluation activities. Figure 1 provides more detail on the different phases of the evaluation. |
| In the introduction, there needs to be more of an explanation of clear constructs. For example what are 'place-based approaches'? How do they differ from alternative models and what is the strength of having a place-based approach. The current explanation that they are different to individual standardised interventions and that they acknowledge social determinants, is quite vague. Real-world examples would be helpful.  Similarly, what is a 'systems change approach', how do they differ from the common orthodoxy? This needs to be bedded down before you go into discussions of complex systems later in the paper. I think you need to do more to take readers along for the ride. Terms like these, unless explained well and integrated well, can start to feel like buzzwords without meaning. | Thank you for this. We agree that explaining these constructs in more detail will be helpful to the reader. We have added to the introduction (from line 61 onwards), including some real-world examples of place-based approaches in the field of young people’s mental health.  “... *These approaches take a variety of different forms, from local partnerships focusing on issue-based change to larger, government-led regeneration in a specific geographical area (6). Place-based approaches usually focus on developing relationships in a community, and they are characterised by partnerships and shared design (7). Examples of such approaches include youth mental health promotion and policy advocacy in Canada (Agenda Gap; 8), co-designed mental health support for 10-16 year olds in the UK (HeadStart; 9) and participatory action research to strengthen mental health services for Aboriginal and Torres Strait Islander young people in Australia (10). Specific collaborators in place-based approaches will vary in relation to goals, but these initiatives all involve long-term work across multiple organisations or actors to create change. Often these approaches act as catalysts for, or explicitly aim to achieve, wider systems change, influencing broader policies and practices and aiming to create population-level impacts (11, 12).”* |
| Similarly 'joined up working' (line 74). | Thank you for this, we have changed this to “improved collaboration” to make it more accessible to readers. |
| In line 94 - isolated to individual-level impacts for whom? For those who participate? And opposed to what? benefits for the whole community?? | Thank you, this was a little unclear but we are trying to highlight the small scale of some of these place-based approaches that aim to reach whole communities. We have clarified this now to say that the effects are “*isolated to a small number of individuals*”. |
| Generally I think you need to give more air time to Kailo as a program. What the program includes, what it doesn't and what it actually aims to do. I got a bit confused because the results section often appeared as if it was evaluating a naturally occurring phenomenon, without out really making clear that Kailo was a pre-defined and carefully developed framework which was delivered in the community. The readers would benefit from a bit more detail about what kailo was trying to achieve, so that the outcomes presented in the evaluation can then be compared against these aspirations. It was also unclear where the role of Kailo begins and ends. Is Kailo just a means of convening existing community members and organisations to have productive discussions about what's best for their communities (i.e. a co-design facilitator)? Or is Kailo also involved in the implementation of these ideas? Does Kailo fund and/or facilitate the proposed community outcomes (e.g., 'Life Skills training for young people'). I appreciate this paper references other papers which describe the Kailo process, but I do think that a bit more detail is needed in this paper to improve its quality as a standalone piece. | Given the scale and complexity of the Kailo framework, we have directed readers to associated papers that provide a detailed description of the Kailo aims and activities. However, it is helpful to know that the summary description for the purposes of this evaluation paper was insufficient/unclear. We have now created a diagram (Figure 2 – at the end of this letter) that provides more detail on the Kailo phases and the funded activities that accompany each phase. |
| Finally, I think more detail is needed about how the young people engaged in the sessions were recruited. Were adolescents with lived experience of mental health issues or poor wellbeing targeted for inclusion - this seems to be an important component of the work. Also, what proportion of those who were approached to participate actually participate? It was mentioned that peers often encouraged each other to come, and some informants were recommended by the Kailo delivery team, but some young people dropped out and never returned. There is of course a real risk when this happens that you get homogenous 'groupthink' from similar peers, and lose out from those who may have benefitted most from the program or provided the most useful feedback based on their lived experience. This feels particularly important when in the next stage of the project you try and understand the distal, long-term population-level impacts, particularly to the local young people most at risk of poor mental health. I think you need to provide more of a rationale in the discussion as to why you did not try and track down those who dropped out of sessions to understand why this occurred and if they had felt comfortable sharing their perspectives during sessions. | Thank you for this helpful comment. We have added more detail to the Methods section on how those leading the Kailo activities in Newham and Northern Devon recruited young people to the co-design sessions.  Specifically, we now say (from line 229 onwards):  *“The Kailo model places a particular emphasis on hearing from young people whose voices are often missing in conversations about the opportunity areas in each site. For the co-design sessions, the Kailo delivery team focused on recruiting young people in these underrepresented groups. Co-design participants were recruited through community partner organisations (e.g. a local charity providing support for autistic young people). Gatekeepers at these organisations who already had existing relationships with young people extended invitations to those they felt might be interested in this work. In total, 44 young people aged 12-25 living in the Kailo pilot sites were recruited to participate in co-design sessions, incorporating diverse groups such as LGBTQIA+ young people, neurodivergent young people, those from white and mixed backgrounds, low-income households, and those from Muslim and Christian faiths.”*  Thank you as well for your comment on speaking to the young people who dropped out of sessions. There was some fluctuation in attendance, often due to changing commitments and schedules. As per our ethical approval for this research, the Kailo site teams had responsibility for recruiting and maintaining communication with young people. After several efforts were made to re-engage young people who stopped attending, we had to treat their lack of response as an opt-out of the research. We agree that it would have been very helpful to speak to these young people and have added the context on this to our Methods section (line 292 onwards):  *“There was some fluctuation in attendance across the co-design sessions in both Northern Devon and Newham. Despite efforts from the organisers to fit sessions around young people, some found that sessions clashed with their work or school commitments, while others did not provide a reason for not attending. As per our ethical approval, the Kailo site teams had responsibility for communicating with young people and, after reasonable efforts were made to re-engage those who stopped attending the sessions, we treated non-response as an opt-out of the research.”*  We have also acknowledged this as a limitation in the Discussion (line 1177):  “However, our study is not without its limitations. *First, we collected data from a subset of community stakeholders and young people, and we were not able to collect data from those who chose not to engage in Kailo at different stages. Understanding why some young people stopped attending co-design sessions would have been particularly useful to inform the development of IPTs.”* |

**Reviewer #2:**

Thank you for taking the time to read and review our manuscript. We were pleased to hear that the content of the article was of interest to you and you appreciated the use of realist evaluation. Thank you as well for your helpful comments, which we respond to in turn below.

| The authors could say more about why a participatory and design-oriented approach is needed if CTC has already demonstrated strong outcomes. I looked at the originating article on Kailo to better understand the overall framework and see strong justification in the 2023 open letter that could be brought into the introduction in this piece. | Thank you for this. We have added to our introduction to include some further justification for the Kailo participatory approach  in the community and potentially improved sustainability. We now say:  “Despite these promising findings, evidence indicates that initial positive impacts of place-based approaches are often not sustained (13) and, in some cases, *their effects are isolated to a small number of individuals* (23). *It is possible too, that a reliance on introducing pre-existing evidence-based programmes may lead to a lack of nuanced understanding of the priorities in a specific local context and miss opportunities to develop local ownership and innovation (24, 25)). As such, participatory approaches that elevate and integrate community voices at all stages of priority setting, design and development are required.”*  We have also added to the Methods section where we provide further summary detail on Kailo (see below) to complement associated references. |
| --- | --- |
| Unless there is another compelling reason, I don’t believe the authors need to frame their study as a developmental evaluation as it is sufficient to note that the study is a realist evaluation of the first two phases of a planned four phased implementation project. In describing this as a developmental evaluation, the reader might expect to see more in the paper on how the results were used to improve the effort as it evolved. | Thank you for highlighting this. Given the scale of the project, we chose to separate out the reporting into two papers. The first is this paper presenting the realist evaluation and the programme theory, and the second is a paper on how the evaluation fed into the Kailo framework delivery and to the development of a detailed theory of change.  We have decided to continue referring to this as a developmental evaluation but have added an explanation for this. At the end of the introduction, we now say:  “*In order to provide sufficient detail on the different elements of the evaluation, our reporting is separated into two papers. This article focuses on surfacing the programme theory, while another describes how the evaluation fed into the delivery of the developing Kailo framework and to the creation of a detailed theory of change.”* |
| The methods would benefit from more operational discussion of the Kailo implementation infrastructure: Who is funder, who are the implementation agents, who monitors implementation alignment with the Kailo framework, and are any funds allocated to implementing plans developed in Phase two. | Thank you for this. Given the scale and complexity of Kailo and its activities, we limited the description to a high-level summary in this paper as it is reported in more detail elsewhere (Hobbs et al., 2023). The appendices (particularly Appendix B) provide information on the different people involved in the delivery of Kailo.  We have now also created a diagram (Figure 2 – at the end of this letter) that provides more detail on the Kailo phases and the funded activities that accompany each phase. |
| In the discussion, the authors note that there were delays in starting the project. I believe the reader would benefit from knowing whether those were true delays (eg funding or a project lead was not able to begin) or simply the natural/normal amount of time it will likely take other sites to adopt this type of community engaged effort? | Thank you for pointing this out. The delays were mainly due to the fact that it was an external team/partnership of academics leading the first iteration of this Kailo framework and it took time to build the necessary relationships in the community. Again, this is something that is explored in more detail in the second paper on the development of the Kailo framework. We have now also added this to the discussion:  Second, despite the intention to follow Kailo throughout one whole lifecycle of the framework (37), delays in initiating the programme in both sites meant we were only able to focus on the first two phases of the framework: Early Discovery and Deeper Discovery and Co-design. These phases represent what had been delivered up to July 2024. *Delays were mainly due to the challenges of research funding timelines and an external team coming in to deliver the framework in Northern Devon and Newham; turnover of community leaders and professionals meant that some of the initial relationships (established before the main application for funding) were lost and it took time to rebuild these connections.* As a result, we were unable to gather data on some of the hypothesised systems outcomes or broader population-level adolescent mental health and wellbeing outcomes. |

Thank you again for your consideration of this manuscript.

Yours sincerely,

Professor Vashti Berry

Associate Professor in Prevention Science

Department of Public Health and Sports Science

University of Exeter Medical School

Figure 2. Overview of Kailo activities and funding
